# Recording from an Identified Neuron Efficiently Reveals Hazard for Brain Function in Risk Assessment

**DOI:** 10.3390/molecules26226935

**Published:** 2021-11-17

**Authors:** Peter Machnik, Stefan Schuster

**Affiliations:** Department of Animal Physiology, University of Bayreuth, D-95440 Bayreuth, Germany; stefan.schuster@uni-bayreuth.de

**Keywords:** chemical safety, risk assessment, risk management, brain function, neuronal function, synaptic balance, synaptic transmission, central nervous system, sensory systems, bisphenol

## Abstract

Modern societies use a continuously growing number of chemicals. Because these are released into the environment and are taken up by humans, rigorous (but practicable) risk assessment must precede the approval of new substances for commerce. A number of tests is applicable, but it has been very difficult to efficiently assay the effect of chemicals on communication and information processing in vivo in the adult vertebrate brain. Here, we suggest a straightforward way to rapidly and accurately detect effects of chemical exposure on action potential generation, synaptic transmission, central information processing, and even processing in sensory systems in vivo by recording from a single neuron. The approach is possible in an identified neuron in the hindbrain of fish that integrates various sources of information and whose properties are ideal for rapid analysis of the various effects chemicals can have on the nervous system. The analysis uses fish but, as we discuss here, key neuronal functions are conserved and differences can only be due to differences in metabolism or passage into the brain, factors that can easily be determined. Speed and efficiency of the method, therefore, make it suitable to provide information in risk assessment, as we illustrate here with the effects of bisphenols on adult brain function.

## 1. Introduction

Many of the products we use every day are made from many different source materials and chemicals that determine critical product features. To improve and to extend these features, or to reduce production costs, a rapidly rising number of new chemicals is being used. In the U.S., for example, an average of two to three new substances have been approved for use every day for the past 23 years [1,2]. However, once released, this diversity of new substances gets part of our lives and can become part of our bodies. Traces of the compounds used in products of daily life, as well as of pesticides and of industrial chemicals, have been detected not only in the environment, but also in human body fluids and tissues [3,4]. Deciding whether the release of any novel substance is potentially harmful for human health and for the environment, therefore, is critical. However, making this decision is challenging and a process that relies, among many other aspects, on practicality and the availability and accuracy of scientific information. We, therefore, start with a brief review of interesting aspects of chemical safety assessment, responsibilities, and strategies with a focus on neurotoxic effects in the vertebrate brain. Standardized in vitro tests and acute/repeated dose toxicity tests applied in animal testing are normally employed to evaluate the potential of a substance to induce neurotoxic effects [5,6,7]. However, significant effects on human brain function can already occur well below the critical levels as determined in established tests of neurotoxicity [7,8]. To assay the effects of chemicals on communication and information processing in the adult brain, suitable in vivo tests are needed. Obviously, such experiments cannot easily be done on humans. Suitable behavioral experiments on animals are conceivable but their connection to specific aspects of neuronal function might not be straightforward and often depend on distributed effects on metabolism, sensory organs, and effectors (e.g., muscles, glands) as well as on neuronal function. Intracellular in vivo recordings, on the other hand, would allow systematically measuring the effects on, for instance, excitability and synaptic transmission. In such an approach a micromanipulator allows positioning a microelectrode into a specific neuron. A suitable amplifier connected to the intracellular and an additional reference electrode then allows one to monitor the membrane potential and its changes when either current is experimentally injected into the neuron (in the so-called ‘current clamp’ mode) or when suitable stimuli cause postsynaptic potentials or action potentials. In the ‘voltage clamp’ mode the membrane current can be measured at various controlled membrane potentials. Such experiments are typically time consuming, can assay only one aspect at a time, and pooling data across individuals requires assumptions on the homogeneity of the neurons they are recorded from. All this is very different in a preparation that we argue here is almost ideal for getting a maximum of information in just one go: Intracellular recording from a single, identified command neuron in the hindbrain of fish that has several useful properties, exhibits on its dendrites synapses that use all transmitter systems known in the vertebrate central nervous system, and that integrates information from various sensory systems. Applied in risk assessment, this approach could supplement the existing efficient tests to quickly and accurately detect effects of substances on a diverse array of specific aspects of brain function. This would–without slowing down product development–allow better decisions in risk assessment to protect our brains. The approach requires the use of fish, but we emphasize that presently no evidence has emerged suggesting that so-called higher or lower animals use different strategies of synaptic communication or of generating resting and action potentials. Once a chemical is found to affect the fish brain all that would be needed is to see whether it also can pass the blood–brain barrier in humans in comparable quantities.

## 2. Standardized Tests Are Used Globally in Risk Assessment

**Figure 1:**
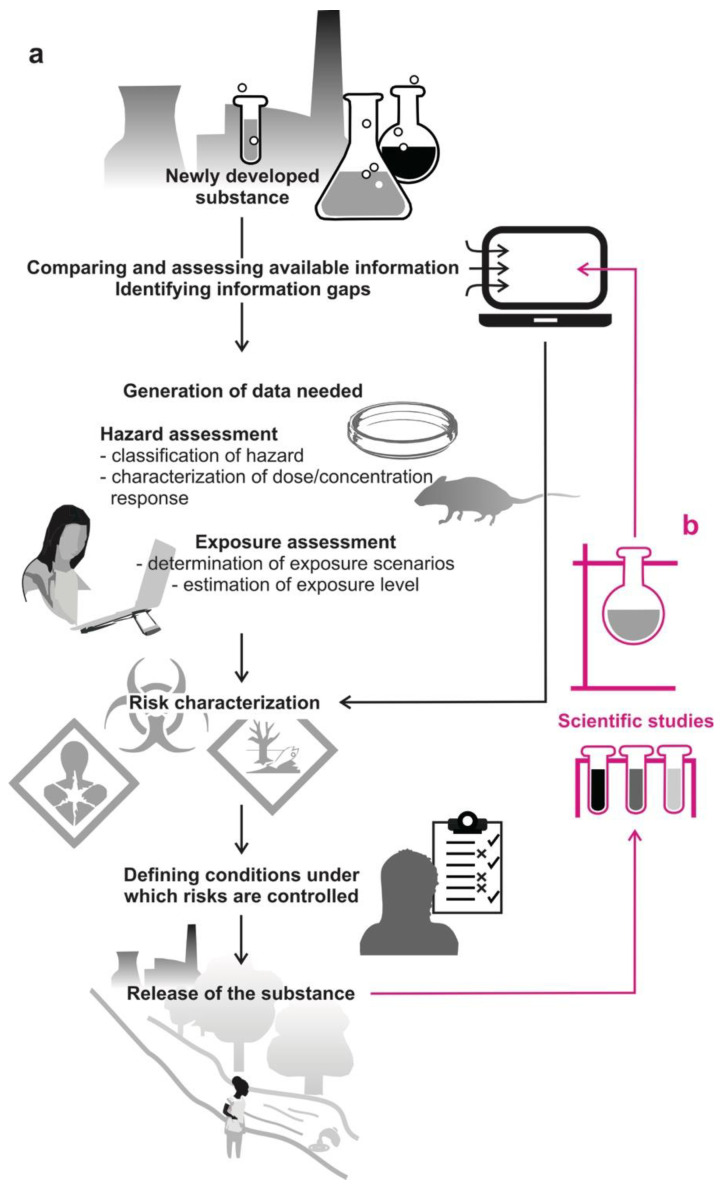
The risk assessment process. (**a**) In most economic areas, manufacturers or importers are accountable for chemical risk assessment in the first place. Based on the available and additionally generated information, they characterize the risk posed by a substance and determine conditions under which the risk appears to be controlled. Subject to compliance with these conditions, the substance may then be used after approval. (**b**) After a newly developed substance is launched, more specific scientific tests occasionally provide results that reveal further hazards that were not known during initial risk assessment and, therefore, could not be taken into account. This would actually necessitate a new assessment of the risk in order to set the no-effect level appropriately.

In most economic areas, newly developed substances are subjected to a risk assessment before being approved for commerce. Figure 1a illustrates the complex process of risk assessment as it applies to hazardous substances in both the U.S. and the EU [9,10], but also applies in many other economic areas [11,12,13,14,15]. Manufacturers, processors, and importers are required to provide information about the new substance they wish to use, taking legislative regulatory requirements into account, and yet keeping speed high and developmental costs low.

Risk assessment comprises two aspects, hazard assessment and exposure assessment. Hazard assessment aims at determining (1) whether a substance is toxic, carcinogenic, mutagenic, and/or reprotoxic and, if so, (2) whether these effects depend on dose/concentration [3,14,16]. Often, hazard is estimated from chemical structure activity relationships, using already available information from studies on similar chemicals [17,18,19]. When no sufficient information exists, then in vitro and/or in vivo tests need to be conducted. In in vitro testing, toxicity is evaluated in specific isolated tissues and the uncritical (‘no effect’) level is then extrapolated to in vivo exposure. In vitro tests provide much more direct information on potential hazards, but are more difficult to control and to interpret. They can also be used as a first step to decide whether more specific testing is needed [7]. The no-effect level determined in animal testing is the highest dose of a substance that does not cause functional disorders or structural changes in experimental animals–normally rodents or fish [20,21]–when exposed or fed daily. Effects on blood composition and weight as well as on the individual organs are examined. The highest dose to which a person can be exposed on a regular everyday basis without any effect on his or her health is then estimated from that, considering additional uncertainty factors and modeling [22].

The second major step in risk assessment, exposure assessment, aims at estimating the dose/concentration of a substance that individuals will be exposed to if the substance would be approved for commerce. This is estimated using exposure models that take production volume, bioaccumulation, potential release when used in a compound, and many additional factors into account and allow for several exposure scenarios and uncertainties [22].

Risks are considered to be controlled when the estimated exposure level does not exceed the no-effect level. Regulatory governmental bodies generally approve the use of the new chemical under those conditions in the respective economic area. More specific testing is not common, and (in most states) not required by law [7,9,10,11,12,13,14,15].

## 3. Beyond Toxicity Tests: The Blind Spots in Risk Assessment

**Figure 2:**
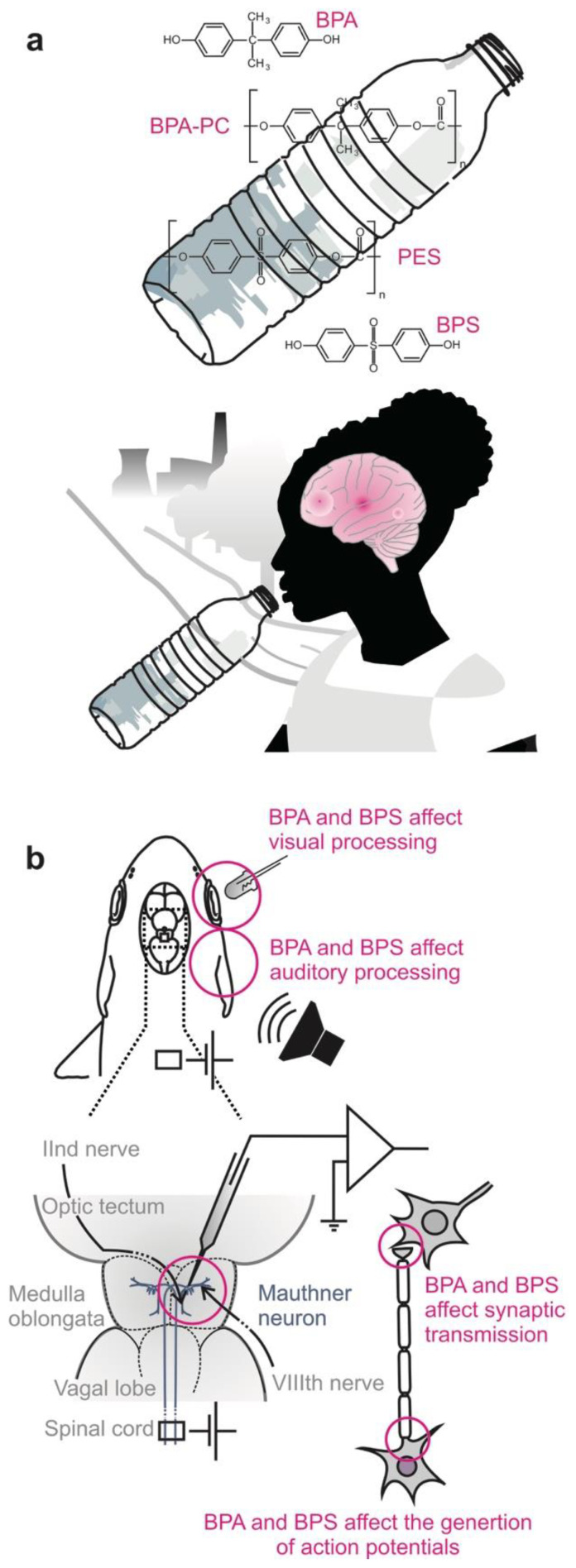
BPA and BPS affect the adult brain. (**a**) BPA is the precursor monomer in the manufacture of polycarbonate (PC), BPS in that of polyethersulfone (PES). Through polycondensation and polymerization of monomers, bisphenols form the backbone of the macromolecular chains of thermoplastics from which they enter the environment and human body. Food packages and plastic bottles are the primary source of human exposure to bisphenols. After having entered the body, they are able to pass the blood–brain barrier and to affect the brain. (**b**) Intracellular in vivo recording in the Mauthner neuron revealed the impact of both BPA and BPS on essential communication functions of neurons and central processing of sensory information in the mature vertebrate brain [54]. One month of exposition to BPA or BPS in environmentally relevant concentration affected the generation of action potentials in the brain as well as the balance between excitatory and inhibitory inputs to auditory and visual sensory circuits.

As we have tried to emphasize above, systematic testing is not the standard requirement in risk assessment. Although suitable test procedures are often both available and approved for use (e.g., [1]), risk assessment still relies heavily on estimates, predictions, and on modeling based on limited data. The majority of the 86,000 substances in commerce in the U.S., for instance, have not been tested for potential neurotoxic effects [1,2]. Less than 50% of the additives allowed by the U.S. Food and Drug Administration (FDA) to be directly added to food have been tested in any animal feeding experiment [23]. It is, therefore, not surprising that numerous examples show how risk assessment failed to adequately estimate exposure levels, no-effect levels, or even the hazard posed by a substance. The lack of testing means that risks are only detected much later in scientific studies paid for by the public at a time when the substance has already been released and accumulated in the environment (Figure 1b). This can be illustrated with the effects of bisphenol A [BPA; 2,2-bis-(4-hydroxyphenyl)-propane; CAS Registry No. 80-05-7] and bisphenol S [BPS; 4,4′-sulfonyldiphenol; CAS Registry No. 80-09-1] on brain function (Figure 2a). Despite considerable concerns, both substances are still in use in numerous everyday products [24,25]. Among others, BPA and BPS are used in the manufacture of polymeric coatings (i.e., the resin lining of cans) and thermoplastic, a versatile material with a huge range of applications. As individuals we come into contact with products made from thermoplastic, for instance, in food packaging, plastic tableware, drinking bottles, dental composites, contact lenses, and children with toys and baby pacifiers [26,27,28,29,30,31]. Thereby, poly(bisphenol A carbonate) [BPA-PC; CAS Registry No. 24936-68-3] is economically one of the most important polycarbonates. Since 1950, BPA is worldwide one of the most frequently used plastic compounds and one of the highest volume-produced organic chemicals [3,32,33]. From the plastic (and further) products, bisphenols are separated via hydrolysis of ester bonds into aqueous liquids or are leached out as the result of incomplete polymerization [34,35,36,37]. Although quantities of separation are low [38], BPA can nowadays be detected ubiquitously from drinking water to dust and BPS is detectable in aqueous environments [39,40,41,42].

Beneath forming the backbone of BPA-PC, BPA also acts as xenoestrogen and exogenous endocrine-disrupting chemical. It affects hormonal balance and reproductive potential and alters neuronal development and metabolic processes in humans, other mammals, birds, and in so-called lower vertebrates [37,43,44,45,46,47,48,49,50,51,52,53]. Due to its hazardous properties, the scope of application of BPA has been limited in the EU to reduce exposure situations [25] and calls are being made for safer alternatives [54,55]. Although bisphenols also leach into the environment, humans appear to be exposed to bisphenols primarily through food packaging and contaminated food (Figure 2a) [24,38,42,56,57]. However, due to the assumption that especially infants are affected by BPA exposure, BPA has been banned in many countries from use in infant feeding bottles (e.g., [38,58]). Emerging concerns about the use of BPA led to the use of hydroxy compounds other than BPA in the manufacture of thermoplastic. Thereby, BPS has become one of the main substitutes of BPA in products labeled to be BPA-free, suspecting that BPS, which is similar to BPA in chemical structure, is a safer alternative. However, a lack of data should not mean safer [59]. Correspondingly, concerns against the use of BPS are rising with the number of studies also revealing detrimental effects of BPS [30,42,49,52,60,61]. Using an in vivo approach, we recently showed that BPA and its main substitute BPS both affect neuronal and synaptic functions in the adult vertebrate brain [54]. Thereby, exposure to small quantities of these plastic compounds over limited time interfered with signal transmission between nerve cells (Figure 2b). The effects of BPA and BPS exposure on neurons, thus, extend to a variety of specific aspects of neural communication in adult vertebrates and are not limited to developing vertebrates as previously assumed and are causal for the banning of BPA not in general, but solely from certain infant products.

Why was this not yet known about compounds that are used in huge quantities and in everyday products? In our opinion, there are three major reasons why hazard assessment completely failed to reveal the danger posed by BPA and BPS to the adult vertebrate brain. (1) Animal tests showed that bisphenols were not directly toxic and were quickly metabolized [62,63]. Since only small quantities of bisphenols are separated from plastic products and cans, they were, therefore, considered safe [32,38] and even consuming them in food is still assumed to be safe [38]. However, this assessment relies on the standard tests as usually used in hazard assessment and fails to take potentially more subtle hazards into account. The strong effects of BPA and BPS on essential communication functions and signal transmission in the adult vertebrate brain could never have been predicted from toxicity tests. Detecting them required a specific approach, the intracellular in vivo recording in a suitable preparation [54]. (2) A battery of potent in vitro assays exists that reveal the effects of substances on neuronal development [7]. However, for the adult brain, in vitro tests cannot take the ability of the brain into account to compensate for many disturbances, and judging the actual in vivo effects can be difficult [64]. Moreover, in vitro studies can obviously not assess the effects of a chemical on the integration of information from various sources distributed over the brain. (3) Behavioral examination works well to reveal the effects of chemicals on developing vertebrates [7,53,60]. However, it is difficult to infer where, specifically, the chemical acts. In contrast to infants or larvae, using adult vertebrates runs into many difficult problems such as not to confound life history effects from the effects of the chemical that is to be examined.

Taken together, determining an adequate no-effect level of a substance on normal brain function, as desirable as it would seem, is an enormous challenge, both to science and to the companies that wish to improve a product.

## 4. Opportunities in Chemical Risk Assessment Arising from an Identified Neuron in Fish

To reveal the effects of BPA and BPS on neuronal signaling in mature vertebrate brains, we recorded from an identified neuron in the central nervous system of fish: the Mauthner neuron (MN). Using it to test the effects of chemicals emerged from a study in which it successfully allowed us to assess the effects of various anesthetics [65]. The MN combines a number of features that make it ideal to quickly and accurately detect the effect of chemicals on a range of fundamental aspects of neuronal communication in the intact adult brain. Recording from intact brains thereby is decisive: The brain uses a range of powerful homeostatic mechanisms that could act in vivo to potentially compensate any effects a substance might have in vitro on isolated neurons [44,64,66,67,68]. Therefore, it requires experiments in the intact brain to test whether the effects seen in vitro are compensated or not.

In the following we give a brief overview of what makes the MN particularly interesting and efficient for assessing the effects of chemicals on adult brains, but we also stress possible limitations of the approach.

**Figure 3:**
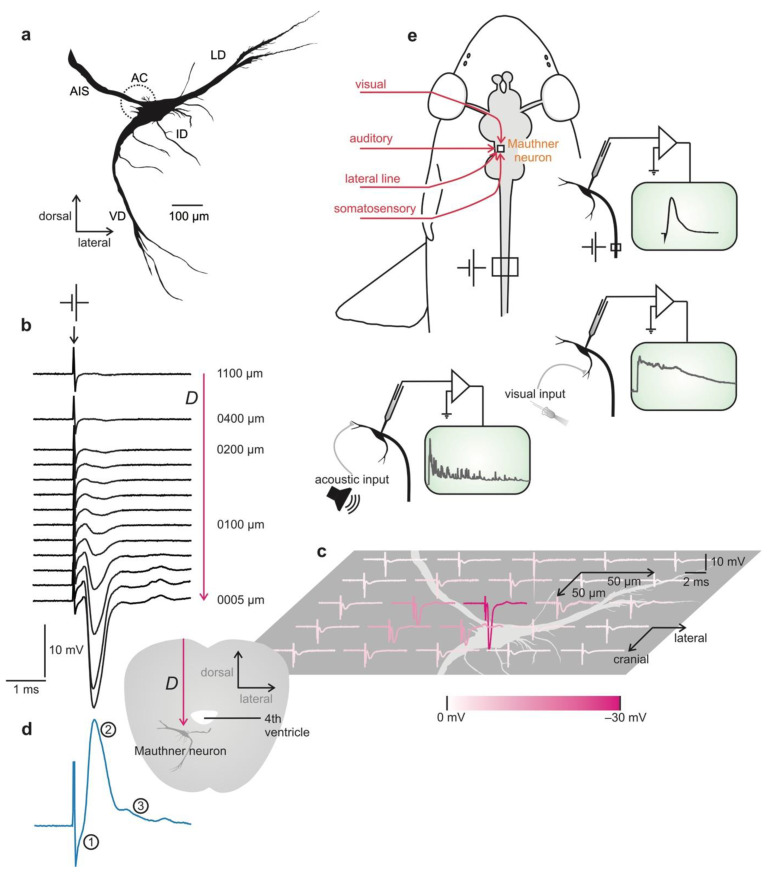
Brief overview of features that make the Mauthner neuron an interesting model for studying effects of chemical exposure on neural function in the adult brain. (**a**) Reconstruction of soma and axonal initial segment (AIS) of the goldfish Mauthner neuron (MN) in coronal view (as seen looking along the fish’s longitudinal axis) (ID = inferior dendrites; LD = lateral major dendrite; VD = ventral major dendrite). The MN is an identified neuron that is easy to localize in the fish brain and to record from. Its axon hillock is surrounded by an associated structure, the axon cap (AC; indicated by dashed circle). When the MN is activated, the axon cap generates an extracellular negative field potential, which has its maximal amplitude in the center of the AC, but can be detected even from distance. This can be used in vivo to guide a recording electrode to the MN soma. (**b**) Direct approach from the medullary surface to the location of one of the two MNs in a goldfish. *D* indicates the distance of the recording electrode from Mauthner soma. (**c**) Field potential amplitude at the depth of the goldfish MN (about 1.1 mm under the surface of the medulla). Magenta gradations indicate the amplitude of the negative spike at respective recording position. Position and orientation of the MN slightly below this plane is indicated in light gray to give an impression of its enormous size. (**d**) After reaching the field potential maximum, the MN regularly can be penetrated by moving the recording electrode a bit further into the brain. It can be identified by several unique characteristics of its antidromically evoked action potential (in blue): (1) extremely short latency between spinal cord stimulation and onset of the action potential, (2) absence of an overshoot and (3) of an undershoot (scaling as in (**b**)). (**e**) shows potential stimulation sides to affect the membrane potential of the MN in goldfish. After establishing intracellular recording, the effects of chemical exposure on neuronal function, action potential generation, synaptic signal transmission, central processing of sensory information, and sensory systems can be determined: Only stimulation (indicated here for antidromic and visual and acoustic stimulation) has to be changed, but recording is kept.

(1) The neuron allows pooling data assembled over time in different individuals. The reticulospinal MN is a so-called identified neuron. Each fish has only two such neurons that can be identified from one fish to the next. Such a situation is rare in the vertebrate nervous system, but offers the enormous advantage that data specifically from this cell can be accumulated in separate experiments performed on different individuals.

(2) Although located deep in the hindbrain, the neuron can easily be localized without visual guidance. To see this intriguing and useful point, it is worth taking a more detailed look (Figure 3). The cell bodies of the two MNs are located in the medulla oblongata, one in the left hemisphere, the other in the right. Figure 3a shows the MN soma and the initial segment of its axon. The axon then continues, crosses the midline, and runs down the entire spinal cord. The trick to localize the soma of the MN in the brain is to stimulate the spinal cord of the fish with sufficiently strong electrical pulses. These will cause action potentials in the large axons of the MNs and these will run toward the somata, a direction called ‘antidromic’. This, in turn, will then cause an extracellular negative field potential that emerges from a complex structure (the ‘axon cap’) surrounding the axon hillock and the axonal initial segment of the MN [69]. This field potential can be detected far away from the MN even at the surface of the brain and can be used to guide a recording electrode to the MN (Figure 3b,c): In the place the field is maximal, the MN can be penetrated, allowing us to directly record, among many other aspects, the antidromically elicited specific MN action potential that allows us to individually identify the MN in vivo (Figure 3d). When recording from the MN is established, all further investigation can begin (Figure 3e).

(3) The Mauthner neuron is gigantic in size, making long-term recording easy. As illustrated in Figure 3a, the cell body of a goldfish MN is unusually large for a neuron in the central nervous system. Its soma can have a diameter of up to 100 µm. This is important to stress in the present context because it greatly simplifies placing a recording electrode in the cell for intracellular recording. Moreover, the large size is also of importance in ensuring impressive stability of the recordings for hours [70], which easily allows running a number of experiments (detailed below) to characterize various fundamental features of neuronal communication.

(4) A great number of crucial features can be determined in one go. The most important point in judging the efficiency of recording from the MN rests in three remarkable properties of the cell. First, as a true command neuron that drives life-saving escape starts [71], the MN integrates information from diverse sensory systems. This allows using a variety of sensory stimuli, recording the responses of the MN to them, and to work out this way the effect of a chemical on each given sensory channel. Second, the antidromic stimulation introduced above allows both action potential generation and processing to be studied in great detail. Third, the existence of mixed synapses allows a particular elegant characterization of the effects of chemicals on the signal transmission of both electrical and chemical synapses. This is easy to appreciate: The action potential travels through the electrical synapses into the presynaptic region, depolarizing it and thus causing transmitter release that is–unusually–produced by an antidromic action potential, but not by sensory input. The effect of this transmitter release on the MN can then easily be recorded. It provides extremely valuable information of synaptic transmission.

Finally, and most important to stress, all that is needed to obtain the various pieces of evidence is to switch from one stimulus to another, i.e., stimulating the axon antidromically, giving visual stimuli, and giving acoustic stimuli. During switching stimulation, the intracellular recording always remains in place and simply records the various responses. This possibility is the key to making the MN preparation so extremely efficient with a large variety of crucial aspects determined in just 2 h of experimentation.

(5) The system allows running experiments in accordance with the 3R principles of animal experimentation. We have previously discovered that even small effects on neuronal function could reliably be detected from measurements taken in groups of only three experimental fish [65]. This not only speeds up data acquisition, but strongly reduces the number of experimental animals needed, as required. Moreover, we have established and tested protocols that allow antidromic and sensory stimulation in surgically anesthetized experimental animals [65] to meet the guidelines and regulations of the animal protection law that are in place in many countries.

In the light of these unusual advantages, one crucial question remains: How relevant would findings obtained in a neuron of the goldfish brain be for human brains? This question comprises mostly two aspects.

First, all evidence available so far suggests that neurons across the animal kingdom basically use the same principles to generate electrical excitability, to transfer information through chemical and electrical synapses and even to adjust synapses during learning. In fact, the basic mechanisms of the action potential have been uncovered in squids, the mechanisms of synaptic transmission have been characterized in frogs and squids, and fundamental views on what changes when we learn have been obtained in a sea slug. This is also the basis for why fish models are well established to, for example, examine specific aspects in neurological disorders in humans [72,73,74,75,76]. Specifically, the MN has already led to major fundamental discoveries, such as the first evidence for axonal protein synthesis, for long-term depression in electrical synapses, consequences of synaptic spillover, or the functional importance of field potentials, to name just a few [77]. Moreover, it is well established [78] that the synaptic inputs into the two major dendrites of the MN (Figure 3a) use all transmitter systems known in the vertebrate brain. Hence, the MN is not only uniquely suited to examine in vivo the impact of chemical exposure on neuronal function but effects found using a MN-based assay should be taken particularly seriously. In case that a given chemical does not show any effect in the MN-based assay, this would mean that it does not affect the sensory cells (photoreceptors, hair cells), the associated neurons in sensory processing (amacrine cells, bipolar cells, horizonal cells, ganglion cells in the retina), neurons in the optic tectum, and all synapses that convey acoustic and visual inputs to the MN. The assay is, thus, not only a survey of the properties of one single neuron but samples effects across several regions of the brain. Additionally, the absence of any effect is thus valuable information, although it is not possible to strictly rule out that some brains or some neuron types differ in sensitivity from all neurons that would be affected in the MN assay. Here, just as in any other good assay, risk assessment also needs the same responsible modelling.

Second, what can be different between the goldfish and human brains is the net accumulation rates of a given chemical in the brain. This depends on the permeability of the blood–brain barrier to the substance but also on the rate at which the chemical is metabolized and/or excreted. Specifically for the bisphenols, we suggest that there should not be major differences between goldfish and humans. This view is based on the similarity of bisphenols with estrogens that are designed to pass the blood–brain barrier so that they exert their many effects on diverse neurons that are equipped with receptors. However, the situation could be different for other chemicals and this would make it advisable to take a two-step approach: If the MN goldfish preparation produces alerting results, then the actual accumulation rate of that substance within the goldfish brain over the experimental period could easily be determined in a subsequent second step to see if similar concentrations could reasonably occur in the human brain.

## 5. Conclusions

How can we do better in assessing the exposure risk for the adult vertebrate brain in chemical risk assessment? Beneath testing the neurotoxicity of a substance, effects of chemical exposure on neuronal functionality and communication and on central information processing should also be taken into account. We argue that the MN preparation in the goldfish poses unusual chances for a rapid, comprehensive, and accurate characterization of the impact of novel substances on diverse key aspects of neuronal function in the intact adult brain. A dedicated lab with three experimenters could accurately characterize a novel substance in 2 days and, so, quickly provide urgently needed, detailed information without unduly slowing down the process of finding useful and safe chemicals.

## Sample Availability

Not available.

## References

[1] EPA Guidelines for Neurotoxicity Risk Assessment. https://www.epa.gov/sites/default/files/2014-11/documents/neuro_tox.pdf.

[2] EPA (2021). About the TSCA Chemical Substance Inventory. https://www.epa.gov/tsca-inventory/about-tsca-chemical-substance-inventory.

[3] Vogel S.A. (2008). From the “dose makes the poison” to the “timing makes the poison”: Conceptualizing risk in the synthetic age. Environ. Hist..

[4] Koch H.M., Calafat A.M. (2009). Human body burdens of chemicals used in plastic manufacture. Phil. Trans. R. Soc. B.

[5] Harry G.J., Billingsley M., Bruinink A., Campbell I.L., Classen W., Dorman D.C., Galli C., Ray D., Smith R.A., Tilson H.A. (1998). In vitro techniques for the assessment of neurotoxicity. Environ. Health Persp..

[6] Coecke S., Goldberg A.M., Allen S., Buzanska L., Calamandrei G., Crofton K., Hareng L., Hartung T., Knaut H., Honegger P. (2007). Workgroup report: Incorporating in vitro alternative methods for developmental neurotoxicity into international hazard and risk assessment strategies. Environ. Health Persp..

[7] Bal-Price A., Pistollato F., Sachana M., Bopp S.K., Munn S., Worth A. (2018). Strategies to improve the regulatory assessment of developmental neurotoxicity (DNT) using in vitro methods. Tox. Appl. Pharm..

[8] Maffini M.V., Neltner T.G. (2015). Brain drain: The costs of neglected responsibilities in evaluating cumulative effects of environmental chemicals. J. Epidemiol. Community Health.

[9] ECHA Guidance on Information Requirement and Chemical Safety Assessment. Part A: Introduction to the Guidance Document. https://echa.europa.eu/documents/10162/13643/information_requirements_part_a_en.pdf/4d25d209-00a8-4a1b-97e5-5adae231b205.

[10] EPA (2021). Human Health Risk Assessment. https://www.epa.gov/risk/human-health-risk-assessment.

[11] Nabholz J.V. (1991). Environmental hazard and risk assessment under the United States toxic substances control act. Sci. Total Environ..

[12] Eisler R. (2000). Handbook of Chemical Risk Assessment.

[13] Bodar C., de Bruijn J., Vermeire T., van der Zandt P. (2002). Trends in risk assessment of chemicals in the European Union. Hum. Ecol. Risk Assess..

[14] Van Leeuwen C.J., Vermeire T.G. (2007). Risk Assessment of Chemicals: An Introduction.

[15] Kim M.-U., Shin S., Byeon S.-H. (2015). Comparison of chemical risk assessment methods in South Korea and the United Kingdom. J. Occup. Health.

[16] Jansen T., Claassen L., van Kamp I., Timmermans D.R.M. (2020). ‘All chemical substances are harmful.’ public appraisal of uncertain risks of food additives and contaminants. Food Chem. Tox..

[17] Van Leeuwen K., Schultz T.W., Henry T., Diderich B., Veith G.D. (2009). Using chemical categories to fill data gaps in hazard assessment. SAR QSAR Environ. Res..

[18] Schultz T.W., Amcoff P., Berggren E., Gautier F., Klaric M., Knight D.J., Mahony C., Schwarz M., White A., Cronin M.T.D. (2015). A strategy for structuring and reporting a read-across prediction of toxicity. Reg. Tox. Pharm..

[19] Escher S.E., Kamp H., Bennekou S.H., Bitsch A., Fisher C., Graepel R., Hengstler J.G., Herzler M., Knight D., Leist M. (2019). Towards grouping concepts on new approach methodologies in chemical hazard assessment: The read-across approach of the EU-ToxRisk project. Arch. Tox..

[20] ECETOC Framework for the Integration of Human and Animal Data in Chemical Risk Assessment. https://www.ecetoc.org/wp-content/uploads/2014/08/ECETOC-TR-104.pdf.

[21] Embry M.R., Belanger S.E., Braunbeck T.A., Galay-Burgos M., Halder M., Hinton D.E., Léonard M.A., Lillicrap A., Norberg-King T., Whale G. (2010). The fish embryo toxicity test as an animal alternative method in hazard and risk assessment and scientific research. Aqua. Tox..

[22] Ciffroy P., Tediosi A., Capri E. (2018). Modelling the Fate of Chemicals in the Environment and the Human Body.

[23] Neltner T.G., Alger H.M., Leonard J.E., Maffini M.V. (2013). Data gaps in toxicity testing of chemicals allowed in food in the United States. Reprod. Tox..

[24] EPA (2010). Bisphenol A Action Plan. https://www.epa.gov/sites/default/files/2015-09/documents/bpa_action_plan.pdf.

[25] ECHA Bisphenol A. https://www.echa.europa.eu/web/guest/hot-topics/bisphenol-a.

[26] Berge T.L.L., Lygre G.B., Jönsson B.A.G., Lindh C.H., Björkman L. (2017). Bisphenol A concentration in human saliva related to dental polymer-based fillings. Clin. Oral Investig..

[27] Wu L.-H., Zhang X.-M., Wang F., Gao C.-J., Chen D., Palumbo J.R., Guo Y., Zeng E.Y. (2018). Occurrence of bisphenol S in the environment and implications for human exposure: A short review. Sci. Total Environ..

[28] Vilarinho F., Sendón R., van der Kellen A., Vaz M.F., Sanches Silva A. (2019). Bisphenol A in food as a result of its migration from food packaging. Trends Food Sci. Technol..

[29] Kovacic A., Gys C., Gulin M.R., Kosjek T., Heath D., Covaci A., Heath E. (2020). The migration of bisphenols from beverage cans and reusable sports bottles. Food Chem..

[30] Thoene M., Dzika E., Gonkowski S., Wojtkiewicz J. (2020). Bisphenol S in food causes hormonal and obesogenic effects comparable to or worse than bisphenol A: A literature review. Nutrients.

[31] Vicente-Martínez Y., Caravaca M., Soto-Meca A. (2020). Determination of very low concentrations of bisphenol A in toys and baby pacifiers using dispersive liquid-liquid microextraction by in situ ionic liquid formation and high-performance liquid chromatography. Pharmaceuticals.

[32] Vogel S.A. (2009). The politics of plastics: The making and unmaking of bisphenol A “safety”. Amer. J. Pub. Health.

[33] GVR Bisphenol A Market Size, Share & Trends Analysis Report by Application (Polycarbonates, Epoxy Resins) by Region, and Segment Forecasts, 2018–2025. https://www.grandviewresearch.com/industry-analysis/bisphenol-a-bpa-market.

[34] Le H.H., Carlson E.M., Chua J.P., Belcher S.M. (2008). Bisphenol A is released from polycarbonate drinking bottles and mimics the neurotoxic actions of estrogen in developing cerebellar neurons. Tox. Lett..

[35] Cooper J.E., Kendig E.L., Belcher S.M. (2011). Assessment of bisphenol A released from reusable plastic, aluminum and stainless steel water bottles. Chemosphere.

[36] Hoekstra E.J., Simoneau C. (2013). Release of bisphenol A from polycarbonate—A review. Crit. Rev. Food Nutr..

[37] Giulivo M., de Alda M.L., Capri E., Barceló D. (2016). Human exposure to endocrine disrupting compounds: Their role in reproductive systems, metabolic syndrome and breast cancer. A review. Environ. Res..

[38] FDA Bisphenol A (BPA): Use in Food Contac Application. https://www.fda.gov/food/food-additives-petitions/bisphenol-bpa-use-food-contact-application.

[39] Arnold S.M., Clark K.E., Staples C.A., Klecka G.M., Dimond S.S., Caspers N., Hentges S.G. (2013). Relevance of drinking water as a source of human exposure to bisphenol A. J. Expo. Sci. Environ. Epidemiol..

[40] Michalowicz J. (2014). Bisphenol A—Sources, toxicity and biotransformation. Environ. Tox. Pharmacol..

[41] Fang Z., Gao Y., Wu X., Xu X., Sarmah A.K., Bolan N., Gao B., Shaheen S.M., Rinklebe J., Ok Y.S. (2020). A critical review on remediation of bisphenol S (BPS) contaminated water: Efficiency and mechanisms. Crit. Rev. Environ. Sci. Technol..

[42] Catenza C.J., Farooq A., Shubear N.S., Donkor K.K. (2021). A targeted review on fate, occurrence, risk and health implications of bisphenol analogues. Chemosphere.

[43] Cobellis L., Colacurci N., Trabucco E., Carpentiero C., Grumetto L. (2009). Measurement of bisphenol A and bisphenol B levels in human blood sera from healthy and endometriotic women. Biomed. Chromat..

[44] Zhou R., Bai Y., Yang R., Zhu Y., Chi X., Li L., Chen L., Sokabe M., Chen L. (2011). Abnormal synaptic plasticity in basolateral amygdala may account for hyperactivity and attention-deficit in male rat exposed perinatally to low-dose bisphenol-A. Neuropharmacology.

[45] Flint S., Markle T., Thompson S., Wallace E. (2012). Bisphenol A exposure, effects, and policy: A wildlife perspective. J. Environ. Manag..

[46] Kajta M., Wójtowicz A.K. (2013). Impact of endocrine-disrupting chemicals on neural development and the onset of neurological disorders. Pharm. Rep..

[47] Rochester J.R. (2013). Bisphenol A and human health: A review of the literature. Repro. Tox..

[48] Heindel J.J., Newbold R., Schug T.T. (2015). Endocrine disruptors and obesity. Nat. Rev. Endocrinol..

[49] Qiu W., Zhao Y., Yang M., Farajzadeh M., Pan C., Wayne N.L. (2016). Actions of bisphenol A and bisphenol S on the reproductive neuroendocrine system during early development in zebrafish. Endocrinology.

[50] Carnevali O., Notarstefano V., Olivotto I., Granziano M., Gallo P., Di Marco Pisciottano I., Vaccari L., Mandich A., Giorgini E., Maradonna F. (2017). Dietary administration of EDC mixtures: A focus on fish lipid metabolism. Aqua. Tox..

[51] Sadoul B., Birceanu O., Aluru N., Thomas J.K., Vijayan M.M. (2017). Bisphenol A in eggs causes development-specific liver molecular reprogramming in two generations of rainbow trout. Sci. Rep..

[52] Qiu W., Liu S., Yang F., Dong P., Yang M., Wong M., Zheng C. (2019). Metabolism disruption analysis of zebrafish larvae in response to BPA and BPA analogs based on RNA-Seq techniques. Ecotox. Environ. Safety.

[53] Kim S.S., Hwang K.-S., Yang J.Y., Chae J.S., Kim G.R., Kan H., Jung M.H., Lee H.-Y., Song J.S., Ahn S. (2020). Neurochemical and behavioral analysis by acute exposure to bisphenol A in zebrafish larvae model. Chemosphere.

[54] Schirmer E., Schuster S., Machnik P. (2021). Bisphenols exert detrimental effects on neuronal signaling in mature vertebrate brains. Comm. Biol..

[55] Trullemans L., Koelewijn S.-F., Scodeller I., Hendrickx T., van Puyvelde P., Sels B.F. (2021). A guide towards safe, functional and renewable BPA alternatives by rational molecular design: Structure-property and structure-toxicity relationships. Polym. Chem..

[56] Kang J.H., Kondo F., Katayama Y. (2006). Human exposure to bisphenol A. Toxicology.

[57] Vandenberg L.N., Hauser R., Marcus M., Olea N., Welshons W.V. (2007). Human exposure to bisphenol A (BPA). Repro. Tox..

[58] EC Commission Directive 2011/8/EU. https://eur-lex.europa.eu/LexUriServ/LexUriServ.do?uri=OJ:L:2011:026:0011:0014:EN:PDF.

[59] Ben-Jonathan N., Hugo E.R. (2016). Bisphenols come in different flavors: Is “S” better than “A”?. Endocrinology.

[60] Gu J., Zhang J., Chen Y., Wang H., Guo M., Wang L., Wang Z., Wu S., Shi L., Gu A. (2019). Neurobehavioral effects of bisphenol S exposure in early life stages of zebrafish larvae (*Danio rerio*). Chemosphere.

[61] McDonough C., Guo D.J., Guo T. (2021). Developmental toxicity of bisphenol S in *Caenorhabditis elegans* and NODEF mice. NeuroToxicology.

[62] Fregert S., Rorsman H. (1962). Hypersensitivity to epoxy resins with reference to the role played by bisphenol A. J. Investig. Dermatol..

[63] Knaak J.B., Sullivan L.J. (1966). Metabolism of bisphenol A in the rat. Toxicol. Appl. Pharmacol..

[64] Belle A.M., Enright H.A., Sales A.P., Kulp K., Osburn J., Kuhn E.A., Fischer N.O., Wheeler E.K. (2018). Evaluation of in vitro neuronal platforms as surrogates for in vivo whole brain systems. Sci. Rep..

[65] Machnik P., Schirmer E., Glück L., Schuster S. (2018). Recordings in an integrating central neuron provide a quick way for identifying appropriate anaesthetic use in fish. Sci. Rep..

[66] Davis G.W. (2013). Homeostatic signaling and the stabilization of neuronal function. Neuron.

[67] Keck T., Keller G.B., Jacobsen R.I., Eysel U.T., Bonhoeffer T., Hübener M. (2013). Synaptic scaling and homeostatic plasticity in the mouse visual cortex in vivo. Neuron.

[68] Hu F., Li T., Gong H., Chen Z., Jin Y., Xu G., Wang M. (2017). Bisphenol A impairs synaptic plasticity by both pre- and postsynaptic mechanisms. Adv. Sci..

[69] Furshpan E.J., Furukawa T. (1962). Intracellular and extracellular responses of the several regions of the Mauthner cell of goldfish. J. Neurophysiol..

[70] Machnik P., Leupolz K., Feyl S., Schulze W., Schuster S. (2018). The Mauthner cell in a fish with top-performance and yet flexibly tuned C-starts. II Physiology. J. Exp. Biol..

[71] Hecker A., Schulze W., Oster J., Richter D.O., Schuster S. (2020). Removing a single neuron in a vertebrate brain forever abolishes an essential behavior. Proc. Natl. Acad. Sci. USA.

[72] Best J.D., Alderton W.K. (2008). Zebrafish: An in vivo model for the study of neurological diseases. Neuropsych. Dis. Treat..

[73] Kalueff A.V., Stewart A.M., Gerlai R. (2014). Zebrafish as an emerging model for studying complex brain disorders. Trends Pharmacol. Sci..

[74] Kozol R.A., Abrams A.J., James D.M., Bulgo E., Yan Q., Dallman J.E. (2016). Function over form: Modeling groups of inherited neurological conditions in zebrafish. Front. Mol. Neurosci..

[75] Fontana B.D., Mezzomo N.J., Kalueff A.V., Rosemberg D.B. (2018). The developing utility of zebrafish models of neurological and neuropsychiatric disorders: A critical review. Exp. Neurol..

[76] Shams S., Rihel J., Ortiz J.G., Gerlai R. (2018). The zebrafish as a promising tool for modeling human brain disorders: A review based upon an IBNS symposium. Neurosci. Biobehav. Rev..

[77] Zottoli S.J., Faber D.S. (2000). The Mauthner cell: What has it taught us?. Neuroscientist.

[78] Korn H., Faber D.S. (2005). The Mauthner cell half a century later: A neurobiological model for decision-making?. Neuron.

